# Methane Oxidation and Molecular Characterization of Methanotrophs from a Former Mercury Mine Impoundment

**DOI:** 10.3390/microorganisms3020290

**Published:** 2015-06-23

**Authors:** Shaun M. Baesman, Laurence G. Miller, Jeremy H. Wei, Yirang Cho, Emily D. Matys, Roger E. Summons, Paula V. Welander, Ronald S. Oremland

**Affiliations:** 1 U.S. Geological Survey, Menlo Park, CA 94025, USA; E-Mails: sbaesman@usgs.gov (S.M.B.); lgmiller@usgs.gov (L.G.M.); 2 Department of Earth System Science, Stanford University, Stanford, CA 94305, USA; E-Mails: jhwei@stanford.edu (J.H.W.); yicho@stanford.edu (Y.C.); welander@stanford.edu (P.V.W.); 3 Department of Earth, Atmospheric, and Planetary Sciences, Massachusetts Institute of Technology, Cambridge, MA 02139, USA; E-Mails: ematys@mit.edu (E.D.M.); rsummons@mit.edu (R.E.S.)

**Keywords:** acid mine drainage, methane stable isotopes, methanotrophic bacteria, sterols, hopanoids

## Abstract

The Herman Pit, once a mercury mine, is an impoundment located in an active geothermal area. Its acidic waters are permeated by hundreds of gas seeps. One seep was sampled and found to be composed of mostly CO_2_ with some CH_4_ present. The δ^13^CH_4_ value suggested a complex origin for the methane: *i.e.*, a thermogenic component plus a biological methanogenic portion. The relatively ^12^C-enriched CO_2_ suggested a reworking of the ebullitive methane by methanotrophic bacteria. Therefore, we tested bottom sediments for their ability to consume methane by conducting aerobic incubations of slurried materials. Methane was removed from the headspace of live slurries, and subsequent additions of methane resulted in faster removal rates. This activity could be transferred to an artificial, acidic medium, indicating the presence of acidophilic or acid-tolerant methanotrophs, the latter reinforced by the observation of maximum activity at pH = 4.5 with incubated slurries. A successful extraction of sterol and hopanoid lipids characteristic of methanotrophs was achieved, and their abundances greatly increased with increased sediment methane consumption. DNA extracted from methane-oxidizing enrichment cultures was amplified and sequenced for *pmoA* genes that aligned with methanotrophic members of the *Gammaproteobacteria*. An enrichment culture was established that grew in an acidic (pH 4.5) medium via methane oxidation.

## 1. Introduction

Methane is the second most important anthropogenic “greenhouse” gas in the Earth’s atmosphere, being on a molecule-per-molecule basis a much stronger absorber of infrared energy than CO_2_, but having a much lower tropospheric abundance (1.8 ppm *vs*. 400 ppm) and residence time (~10 years *vs*. 300 years). Nonetheless, large releases of methane from sequestered sources (e.g., gas bubbles in frozen arctic lakes or destabilized gas hydrates) can act as a climatic feedback accelerant by increasing ambient temperatures enough to promote an even greater outward flux of additionally entrapped CO_2_ and CH_4_ [1]. Aside from its chemical destruction in the troposphere by reaction with hydroxyl radicals [2], the other important methane sink term is its consumption as an energy and carbon source by diverse prokaryotes that inhabit anoxic, sub-oxic, and oxic biomes [3]. Hence, upward migration of a methane bubble formed within anoxic sediments must survive transit through this gauntlet in order to be released to the atmosphere, the final barrier being occupied by the well-studied aerobic methane-oxidizing bacteria (methanotrophs). Aerobic methanotrophs have long been an object of scientific fascination owing to their unique biochemical pathways of one-carbon unit oxidation and fixation into cell material (e.g., [4,5,6,7]).

Although aerobic methanotrophs have been known for over a century since the first description by Söhngen [8], it was a series of papers that appeared 45 years ago which gave great impetus to the field by clarifying cultivation requirements and broadly characterizing and classifying 100 novel isolates [9,10,11]. This seminal work along with the many contributions of other researchers over the subsequent decades gave rise to a certain dogma, namely that of obligate methanotrophy, whereby all methane-oxidizing bacteria were capable of growth only upon methane or organic compounds that lacked carbon–carbon bonds (e.g., methanol). Evidence for the possible existence of facultative methanotrophs that could grow on substrates like acetate as well as methane was controversial and contested (e.g., see commentary by Theisen and Murrell [12]) until the discovery of novel species of acetate-utilizing *Methylocella* that inhabit acidic peat bogs [13,14,15]. The idea that methanotrophs could thrive in acidic environments was pursued further with the discovery of the Verrucomicrobia, true methanotrophic extremophiles that inhabit geothermal regions and could grow at pH < 1.0 [16,17,18]. Dunfield [19] has reviewed the full extremophilic spectrum of known methanotrophy, which in addition to thermophiles and acidophiles includes examples from hypersaline and alkaline environments.

The Herman Pit (39°00′ N; 122°39.5′ W) is a large impoundment located on the southeastern shores of Clear Lake, California. It occupies the site of the abandoned Sulfur Bank mercury mine (cinnabar: HgS) active from 1873 to 1957. It is a U.S. Environmental Protection Agency Superfund Site where concern focuses upon the subsurface transport of mercury and other contaminants into local ground-waters as well as into this large recreational lake [20]. The Sulfur Bank has active hydrothermal mineral ore precipitation [21,22] and ebullition of geothermal gases that contain methane [23]. The waters of the Herman Pit itself are strikingly clear, azure-hued, acidic (pH range 2–4), and permeated throughout by hundreds, perhaps thousands, of constant-flow gas seepages (~100 mL·min**^−^**^1^). The surface waters are typically mesothermal (annual range: 11–22 °C), well oxygenated (dissolved O_2_ = 160–218 μM), and the surficial sediments contain mercury (9–44 mg/kg) [24]. 

Wells and Ghioso [21] discounted the occurrence of methane oxidation in the Herman Pit based on observations with boiling hydrothermal systems made by Drummond and Ohmoto [25]. Indeed, Wells and Ghiorso [21] went so far as to make the following blanket statement: “In nature, the oxidation of CH_4_ is probably insignificant because of the sluggish kinetics of the reaction.” Although the Herman Pit may appear boiling due to the above mentioned massive gas ebullition, it is generally mesothermal and well aerated (see above). The above given examples of methanotrophs isolated from extremes of pH, and the detection of methane-oxidizing activity and genes in hot springs [26,27] prompted us to search for methanotrophy in the sediments of the Herman Pit. We now report the presence of methane-oxidation and aerobic methanotrophs in these metal-contaminated sediments, which to our knowledge is a first for detecting this process in an acid mine drainage environment permeated by hydrothermal gases.

## 2. Methods and Materials

### 2.1. Sediment Slurry Incubations

Near-shore surface sediments were collected in March, 2014 using an Ekman grab. The highly porous (“soupy”), orange-colored mud was stored in completely filled mason jars that were kept refrigerated until experiments commenced (within a few days of sampling). Initial experiments were conducted using 3:1 mixtures of the collected lake water: sediment so as to generate dilute slurries after homogenization. Slurries (25 mL) were dispensed into 59 mL serum bottles, crimp-sealed with blue butyl rubber stoppers under air and then injected with 0.3 mL CH_4_ (~1% of gas phase). Slurries were incubated statically in the dark at room temperature (~20 °C), and the gas phase was periodically sampled (after vigorous hand-shaking) by syringe for methane analyses. When the CH_4_ was observed to have been entirely removed, additional CH_4_ and O_2_ (1:2) were added by syringe injection. A series of 5 mL transfers were made from this initial slurry into serum bottles that contained an acidic (pH 4.0) artificial medium originally designed for the cultivation of iron-oxidizing acidophiles [28]. In lieu of air, the gas phases in these incubations contained mixtures with higher oxygen (40% O_2_ and 60% N_2_; 1% CH_4_) to compensate for the absence of gas exchange promoted by shaking. After the first successful transfer, a pH range experiment was conducted in the artificial medium using diluted slurry (volume 5 mL) from a bottle that had achieved successful consumption of several pulsed additions of CH_4_. The pH was poised with additions of either H_2_SO_4_ or NaOH at the following initial values (aerobic experiments): 3.0, 4.5, 5.0, 7.0, 9.0, and 11.0, the conditions received buffer of 20 mM glycine, no addition, malate, MOPS, AMPSO, and CAPS, respectively.

### 2.2. Enrichment Cultures

Culture tubes (25 mL “Balch”) containing 10 mL of the above-given artificial medium [28] adjusted to pH 4.5 and were crimp-sealed under air with blue butyl rubber stoppers and injected with slurry (1 mL) from a CH_4_-consuming serum bottle. Tubes were over-pressurized by injection of 3 mL of an O_2_:CH_4_ mixture (2:1) and dark-incubated at 28 °C in a horizontal orientation with reciprocal shaking so as to facilitate gas exchange. Growth and methane consumption could be sustained for only 2 transfers before petering out. This was alleviated slightly by inclusion of 0.5 g/tube of sterilized montmorillonite, but this line too soon gave out. A shift to the medium for cultivation of Verrucomicrobia [16], adjusted to pH 4.5 and lacking the yeast extract was then applied and used forattempts at continued cultivation. The inoculum (0.5 mL) was from an earlier transfer line in medium [28], which still contained some sediment from the first enrichments.

### 2.3. Analytical

Dissolved CH_4_ was extracted from surface water using an air equilibration technique [29]. Methane/air samples were transferred to rubber-stoppered 10 mL serum bottles [30] for analysis the following day. Gases contained in seep bubbles (including CH_4_, C_2_H_6_, and CO_2_) were collected directly by water displacement and stored in 30 mL serum bottles for several days prior to analysis. Hydrocarbon concentrations were determined using FID-GC [31] while CO_2_ concentrations were determined by TCD-GC [32]. Values of δ^13^CH_4_ and δ^13^CO_2_ were determined by cavity ring-down spectroscopy (CRDS) [33,34] on samples of seep gases diluted in Tedlar bags with UHP nitrogen. Carbon isotopic measurements were made using a Picarro G2101-*i* analyzer sampling at a flow rate of 25 mL/min. The CRDS was calibrated for ^13^CH_4_ using room air and mixed standards provided by isometric instruments (Victoria, BC). Calibration of ^13^CO_2_ was performed with gas derived from acid reaction with NaHCO_3_. The resulting isotopic values were corroborated by analysis of δ^13^CH_4_ of standards by dual inlet mass spectrometry at UC Davis and of δ^13^CO_2_ analysis of NaHCO_3_ powder by combustion-interfaced IRMS at the USGS Stable Isotope Laboratory in Menlo Park, CA.

### 2.4. Lipid Analysis

Lipid extracts from sediment slurries and enrichments were prepared using the method of Welander *et al*. [35] with the following modifications. Approximately 5 g of sediment slurry were re-suspended in 10 mL of 10:5:4 (v:v:v) methanol (MeOH):dichloromethane (DCM):water and sonicated for 1 h. For the sediment CH_4_ enrichments, 20 mL of the sample was centrifuged at 5000× *g* and the supernatant was discarded prior to re-suspension in the MeOH:DCM:water mixture and sonication. The samples were then separated into two phases via the addition of 10 mL DCM and 10 mL water followed by centrifugation at 2800× *g* for 10 min. The organic phase was placed in a new tube and the residual aqueous phase was extracted once more with 10 mL DCM and 10 mL water for a total of two extractions. Following centrifugation, the organic phases were combined and evaporated under a stream of N_2_ gas and the total lipid extracts (TLE) were then dissolved in 2 mL DCM and syringe filtered. To identify the production of C30 hopenes and methylated sterols, acetylated TLEs from each sample were analyzed by high temperature gas chromatography-mass spectrometry (GC-MS/MS) as previously described [36] with the following modifications. Briefly, lipid extracts were analyzed using an Agilent 7890 B Series GC, equipped with a DB5-HT (30 m × 0.25 mm i.d. × 0.125 μm film thickness, Agilent Technologies, Santa Clara, CA, USA) column using helium carrier gas at a constant flow rate of 1.8 mL/min. Two microliters of sample was injected in splitless mode at 360 °C with the following GC program: 100 °C for 2 min, ramp 15° C /min to 360 °C and hold for 28 min, with the GC coupled to a Agilent 5977A Series MSD with a source operating at 230 °C. Acetylated TLEs were also analyzed by liquid chromatography-mass spectrometry (LC-MS) to identify any functionalized hopanoids [37]. Identification of lipid molecules was done using their exact mass and by comparison of the retention time and the mass spectra with published data [38,39].

### 2.5. Phylogenetic Analysis

The PowerSoil DNA Isolation Kit (MoBio Laboratories, Inc., Carlsbad, CA, USA) was used to extract total DNA from 10 mL of enrichment culture after pelleting the cells at 5000× *g* for 10 min. The following primers were used for amplification of pmoA (A189f (GGNGACTGGGACTTCTGG), and A682r (GAASGCNGAGAAGAASGC) [40] and of the 16S rRNA gene (8F (AGA GTT TGA TCC TGG CTC AG) and 1492R (CGG TTA CCT TGT TAC GAC TT) [41]. Amplified bands of the proper size were gel purified and cloned utilizing the TOPO TA cloning kit (Life Technologies, Grand Island, NY, USA). Plasmids from positive clones were isolated using the Qiagen Mini-Prep Plasmid Kit (Qiagen, Valencia, CA, USA). Twenty-one pmoA and thirty-eight 16S rRNA inserts were sequenced using primers specific to the TOPO vector at Elim Biopharm (Hayward, CA). Phylogenetic analysis was performed by the following processes: The pmoA gene sequences and 16S rRNA gene sequences were identified by BLASTX and BLASTN search [42] respectively, via the IMG platform (Integrated Microbial Genomics) (https://img.jgi.doe.gov). The pmoA and 16S rRNA gene sequences of closely related taxa obtained from the GenBank database were aligned using the CLUSTAL Omega [43]. Phylogenetic analysis was performed using the program MEGA 6 [44]. Distance matrices were calculated according to the Jukes and Cantor model [45]. Phylogenetic trees were inferred by neighbor-joining [46] using bootstrap analyses based on 1000 replications.

## 3. Results

### 3.1. Gases

The abundance and composition of the gases collected at the surface of the Herman Pit are given in Table 1. Carbon dioxide was the dominant component of the seep gas, but a significant amount of CH_4_ was also present, and these values fall in line with previous measurements of gases collected in this region [23]. The δ^13^C values for CO_2_ were about 3–5 per mil more ^12^C-enriched than that measured for atmospheric CO_2_ (e.g., [47,48]) but considerably heavier, by about ~10 per mil, than that typically found in plant material (e.g., −20 to −30 per mil; [49]). The δ^13^CH_4_ was strongly ^13^C-enriched, usually indicative of a thermogenic rather than biogenic origin [50], although the process of bacterial methane oxidation could achieve a similar effect of ^13^C-enrichment of the residual, un-oxidized methane (e.g., [29,51]). The trace levels of ethane, together with propane and butane being below limits of detection, further support a predominant biogenic origin for the methane since higher alkanes are usually abundant in natural gases of thermogenic origin, yielding C_1_/[C_2_ + C_3_] ratios well below 100 [52]. Due to the pervasive ebullition of methane-containing seeps, the lakewater concentration of dissolved methane was exceptionally high, being at least two orders of magnitude greater than that determined for the surface waters of methane-rich waters of Mono Lake and Searlesville Lake [30].

**Table 1:** Composition and δ^13^C values of gases collected from Herman Pit seeps and extracted from the aqueous phase.

Component	Concentration	δ^13^C (‰)
CO_2_	95%	−11
CH_4_	5%	−24
C_2_H_6_	10–20 ppm	BD
C_1_/C_2_ + C_3_	>5000	ND
Dissolved CH_4_	400 μM	ND

ND = not determined. BD = below detection.

### 3.2. Sediment Incubations

**Figure 1:**
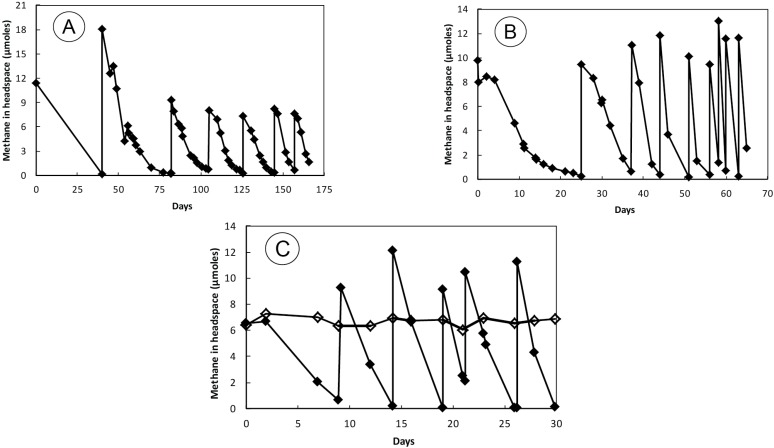
Methane oxidation by unshaken sediment slurries generated from the Herman Pit. (**A**) Initial incubation in lake water from which an aliquot was transferred into an artificial medium (**B**). (**C**) Consumption of methane in Balch tubes incubated with aliquots from the previous incubation (**B**) in a serum bottle. Open diamonds indicate enrichment incubated under air while closed diamonds indicate an enrichment incubated under 40% O_2_.

**Figure 2:**
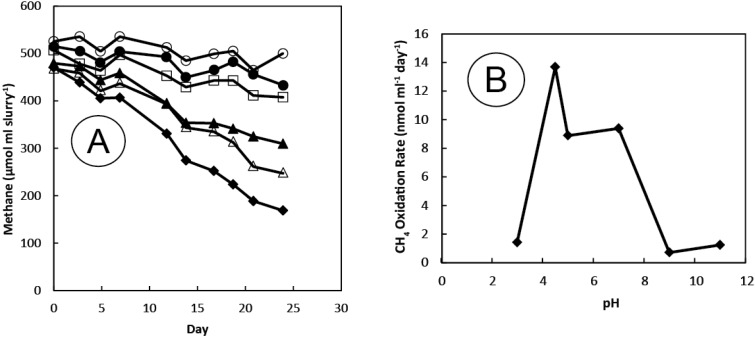
Consumption of methane by sediment slurries incubated at different buffered pH values. (**A**) Symbols for pH: □ (3.0), ♦ (4.5), ▲ (5.0), ∆ (7.0), ● (9.0), ○ (11.0). (**B**) Rates of methane consumption as extrapolated from the slopes of the uptake curves displayed in panel (**A**). Results represent the progress curves for single (*n* = 1) experimental sediment slurry incubations held at the given pH values. The r^2^ values (std. error) of the negative slopes of the regression lines obtained from panel A as applied to panel B were: pH 3: 0.49 (0.31); pH 4.5: 0.99 (0.59); pH 5.0: 0.90 (1.51); pH 7.0: 0.95 (0.82); pH 9.0: 0.20 (0.31); and pH 11.0: 0.37 (0.35).

Sediment slurries consumed CH_4_ over the initial prolonged incubation (~190 days). By Day 40, all of the methane had been removed from the headspace and a complete removal persisted with each of seven subsequent pulsed additions of CH_4_ + O_2_ (Figure 1A). The entire content of this bottle was used for the first round of hopanoid analysis (see below). Transfer of an aliquot of this material into a second serum bottle containing artificial medium resulted in sustained CH_4_ oxidation, and consumption rates increased with the repeated nine pulsed additions of CH_4_ + O_2_ made over a 65 day time course (Figure 1B). This is the slurry line from which a second round of hopanoid analysis was carried out and DNA was extracted for 16S rDNA gene sequences and *pmoA* amplifications. A third sequential transfer of 2 mL of slurry was made into two Balch tubes. Methane was consumed in a tube incubated under a gas mixture rich in oxygen (40% O_2_ + 60% N_2_ + 1% CH_4_) and exhibited similar patterns of CH_4_ oxidation with six pulsed CH_4_ + O_2_ additions made over a 30 day period (Figure 1C). A tube incubated under an air headspace +1% CH_4_, however, did not consume methane. Sediment slurries demonstrated highest methane consumption over a relatively narrow range of pH values, with no activity at pH 11 and only slight activity occurring at pH 3 and 9. Highest rates were observed in the neutral to slightly acidic range (4.5–7.0; Figure 2A). When the progress curves were calculated as rates, the optimum pH for methane oxidation was 4.5, which was the measured pH of Herman Pit water during sampling.

### 3.3. Hopanoid and Sterol Production

**Figure 3:**
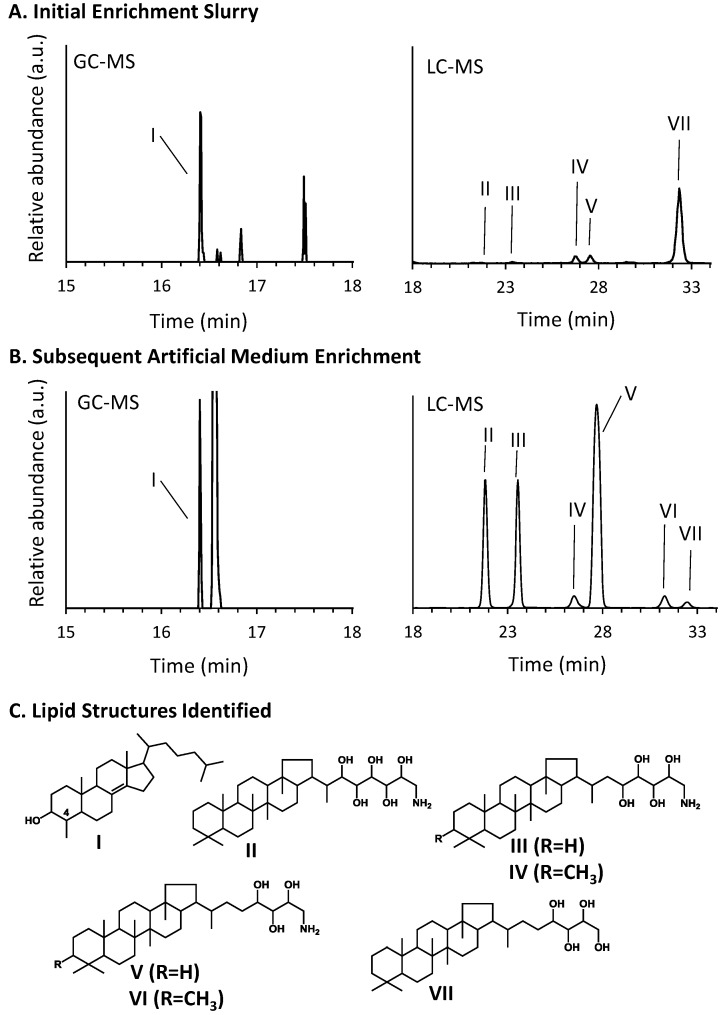
Detection of aerobic methanotroph sterols and hopanoids in Herman Pit enrichment samples. GC-MS extracted ion chromatograms (*m*/*z* 442) showing production of a key aerobic methanotroph sterol and LC-MS combined extracted ion chromatograms (*m*/*z* 830, 772, 786, 714, 728, and 655) showing production of 3-methyl and desmethyl aminohopanoids in (**A**) initial slurry enrichments and in (**B**) subsequent artificial medium incubations. After increased incubation in artificial medium, a significant increase is observed in aminohopanoid production and C-3 methylation indicating an enrichment of aerobic methanotrophs. (**C**) Key sterol and hopanoid structures identified in enrichment samples: (I) 4-methylcholestenol, (II) aminopentol, (III) aminotetrol, (IV) 3-methylaminotetrol, and (V) aminotriol.

Initial sediment slurries incubated in freshwater medium with pulses of methane for approximately 190 days were analyzed for polycyclic lipids characteristic of aerobic methanotrophs. Specifically, we looked for the production of bacteriohopanepolyols (extended hopanoids) with amino modifications at the C-35 position and/or with a methylation at the C-3 position [38] and for the production of C-4 methylated sterols [53]. In these slurries we saw a significant amount of 4-methylcholestenol and traces of the aminohopanoids (Figure 3). As mentioned above, an aliquot of the slurry was transferred to artificial medium and after 65 days of incubation were re-examined for hopanoid production. In this methane-enriched diluted sediment slurry sample, a significant increase in aminopentol, aminotetrol and aminotriol production was observed as well as in increase in 3-methylaminotetrol and 3-methylaminotriol (Figure 3 and Table 2). Typically, aerobic methanotrophs are divided into two phylogenetic classes—the *Gammaproteobacterial* (Type I) and the *Alphaproteobacterial* (Type II) methanotrophs. Both groups are known to produce amino hopanoids but *Alphaproteobacterial* species are only thought to produce aminotetrol and aminotriol and do not methylate these molecules at the C-3 position [35,54]. In addition, 4-methylsterols have only been observed in *Gammaproteobacterial* methanotrophs [53]. Thus, the combination of hopanoid lipids and sterol observed in these sediment slurries and enrichments resembles the profile of a *Gammaproteobacterial* methanotroph rather than *Alphaproteobacterial* species. We do not rule out the possibility that representatives of *Alphaproteobacterial* species are present within the Herman Pit sediments (see below), but only that our incubations, as discerned from the increases in detected select hopanoids/sterols with time (Table 2) is best interpreted that they favored proliferation of Type I over Type II species.

**Table 2:** Hopanoid production measured in Herman Pit enrichments.

Hopanoid Produced	μg hopanoid/μg total lipid extract	
	Initial Slurry	Enrichment Culture
V. Aminotriol	0.2	82
III. Aminotetrol	0.04	30
II. Aminopentol	0.03	32
VI. 3-Methylaminotriol	BD	4
IV. 3-Methylaminotetrol	0.2	4
VII. Bacteriohopanetetrol	2	1

BD = below detection.

### 3.4. Amplification and Sequencing of 16S rRNA and pmoA Genes

**Figure 4:**
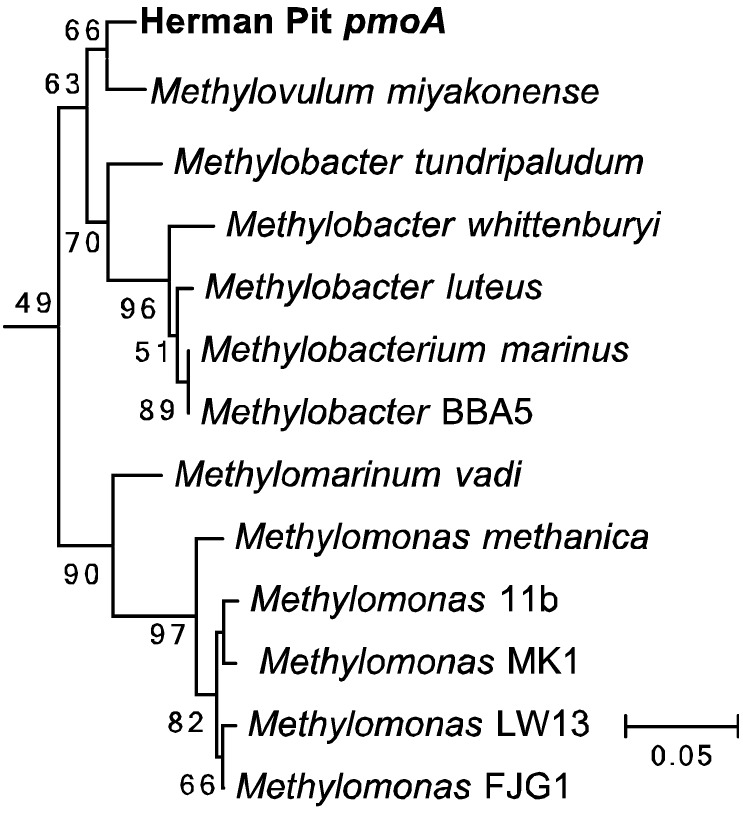
Neighbor-joining tree of the derived amino acid sequence of the *pmoA* gene amplified from the Herman Pit enrichment sample.

**Figure 5:**
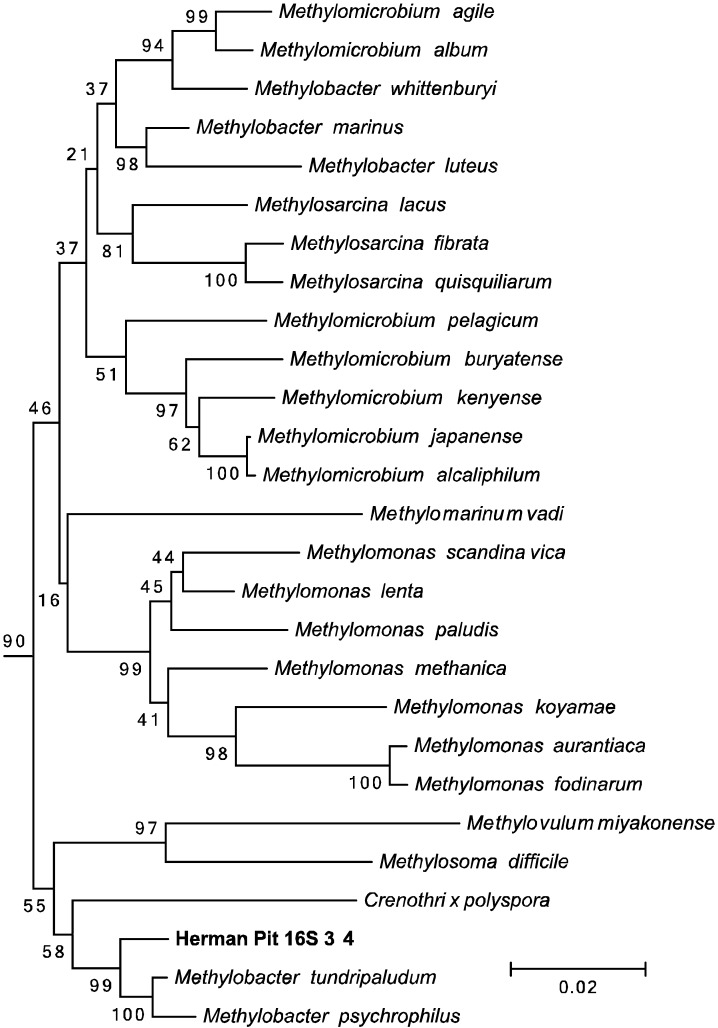
Neighbor-joining tree of one 16S rRNA gene amplified from the Herman Pit enrichment sample demonstrating its close relationship to the *Gammaproteobacterial* class of aerobic methanotrophs.

**Figure 6:**
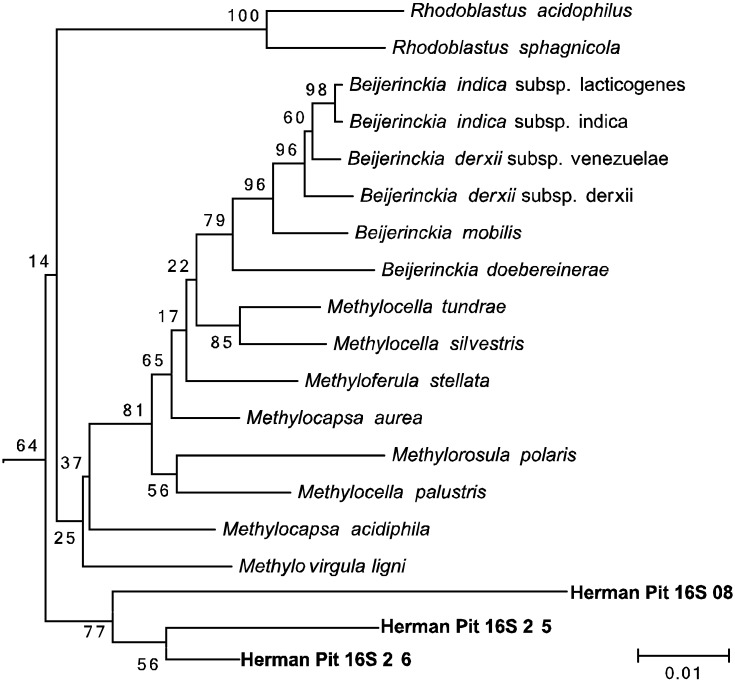
Neighbor-joining tree of three 16S rRNA gene amplified from the Herman Pit enrichment sample demonstrating their close relationship to the *Alphaproteobacterial* class of aerobic methanotrophs.

To determine the identity of the hopanoid producing methanotroph(s) in our enrichments, total DNA was extracted from 10 mL of the same enrichment culture used in the second lipid analysis described above. Degenerate primers were used to amplify the particulate methane monooxygenase (*pmoA)* gene [55] and the 16S rRNA gene. Twenty-one *pmoA* clones were sequenced and all clones contained the same *pmoA* sequence, which clustered within the *Gammaproteobacteria*. This sequence was most closely related to *Methylovolum* (95%–97%) and *Methylobacter* (93%–95%) species (Figure 4). This agreed with the 16S rRNA results, which revealed one methanotroph sequence that fell within the *Gammaproteobacteria Methylobacter* taxa (98.5%) (Figure 5). However, we were also able to amplify the 16S rRNA gene from seven other bacterial species. Four of these sequences clustered within genera not associated with methanotrophy (two *Alphaproteobacteria*, *Phreatobacter* sp. and *Rhizobium* sp., and two *Gammaproteobacteria*, *Dyella* sp. and *Rhodanobacter* sp.) The other three 16S sequences were most closely related to *Alphaproteobacteria* methanotrophs or methylotrophs, *Methylocapsa* (97.3%) and *Methylovirgula* (95.6%) (Figure 6). Thus, acidiphilic or acidotolerant aerobic methanotrophs from both the *Alpha* and *Gammaproteobacteria* appear to be present in Herman Pit, and were carried with CH_4_-incubation of slurries. However, based on the lipid profile observed and the amplification of *pmoA* only from the *Gammaproteobacteria*, our culturing methods seem to have enriched primarily a *Gammaproteobacterial* methanotroph, which previous studies have shown can be difficult to enrich from acidic environments [56].

### 3.5. Amplification and Sequencing of 16S rRNA and pmoA Genes

To determine the identity of the hopanoid producing methanotroph(s) in our enrichments, total DNA was extracted from 10 mL of the same enrichment culture used in the second lipid analysis described above. Degenerate primers were used to amplify the particulate methane monooxygenase (*pmoA)* gene [55] and the 16S rRNA gene. Twenty-one *pmoA* clones were sequenced and all clones contained the same *pmoA* sequence, which clustered within the *Gammaproteobacteria*. This sequence was most closely related to *Methylovolum* (95%–97%) and *Methylobacter* (93%–95%) species (Figure 4). This agreed with the 16S rRNA results, which revealed one methanotroph sequence that fell within the *Gammaproteobacteria Methylobacter* taxa (98.5%) (Figure 5). However, we were also able to amplify the 16S rRNA gene from seven other bacterial species. Four of these sequences clustered within genera not associated with methanotrophy (two *Alphaproteobacteria*, *Phreatobacter* sp. and *Rhizobium* sp., and two *Gammaproteobacteria*, *Dyella* sp. and *Rhodanobacter* sp.) The other three 16S sequences were most closely related to *Alphaproteobacteria* methanotrophs or *methylotrophs, Methylocapsa* (97.3%) and *Methylovirgula* (95.6%) (Figure 6). Thus, acidiphilic or acidotolerant aerobic methanotrophs from both the *Alpha* and *Gammaproteobacteria* appear to be present in Herman Pit, and were carried with CH_4_-incubation of slurries. However, based on the lipid profile observed and the amplification of *pmoA* only from the *Gammaproteobacteria*, our culturing methods seem to have enriched primarily a *Gammaproteobacterial* methanotroph, which previous studies have shown can be difficult to enrich from acidic environments [56].

### 3.6. Enrichment Cultures

Cultivation of methanotrophs was attempted in Balch tubes incubated under O_2_-enriched atmospheres as shown in Figure 2C. However, although increased turbidities (e.g., A_600_ = 0.3) were achieved over time with repeated addition and consumption of methane as occurred with sediment slurries, the ability to sustain a stable liquid enrichment culture capable of consistent sub-transfers into media of pH ~4.5 proved problematical. Methane oxidation in these transfers could only be sustained beyond one, or at most two, transfers if a small portion of the sediment solids contained in some of the original enrichments was added to the tubes. This was a situation reminiscent of the enhancement of methane oxidation by cultivars made by inclusion of clay minerals from Lake Erie [57]. Initially cells were evenly dispersed into the aqueous phase, but as growth progressed they clumped together forming short (2–3 mm) string-like clusters.

## 4. Discussion

Our results with incubated sediment slurries show that aerobic methane oxidation can be readily elicited with Herman Pit sedimentary material (Figure 1A). The continued additions of methane were followed by its complete removal. This was coupled with the fact that drawdown rates increased over time in a saw-tooth patterns that are characteristic of a microbial process whereby cell numbers increase with re-supply of the depleted substrate. The fact that the process could be sub-transferred twice sequentially into fresh media, thereby further diluting the sediment component, underscores this interpretation (Figure 1B,C). Hence, the statement by Wells and Ghioso [21] suggesting methane oxidation being too “sluggish” and hence not a major sink for dissolved oxygen in the Herman Pit can be challenged. Whether methane oxidation by these sediments occurs at significant rates under *in situ* conditions and contributes to depletion of dissolved oxygen of the impoundment water relative to oxidation of Fe(II) and H_2_S cannot be directly extrapolated from experiments of this nature. It would require further effort using intact sediment sub-cores and water samples taken from discrete depths, facilitated by usage of radiotracers (e.g., ^14^CH_4_), procedures that have been well-worked out by a number of past investigations made in other fresh waters (e.g., [58,59,60,61,62]). Aside from oxygen consumption, the presence of a viable population of methanotrophs in the Herman Pit system may contribute to the relatively ^13^C-enriched and ^13^C-depleted values of δ^13^CH_4_ and δ^13^CO_2_, respectively, as was observed in the collected seep gases (Table 1).

The sediment slurries showed enhanced methane oxidation activity at a mildly acidic pH (4.5), which was about the same value as the lake water itself (Figure 2A,B). The general absence of activity at the pH extremes, but the clearly discernable uptake at circum-neutral pH would suggest that the sediments harbor mildly acidophilic methanotrophs as opposed to just acid-tolerant strains. Certainly they are not comparable to the unusual strains of Verrucomicrobia isolated from and adapted to environments that are highly acidic (pH ≤ 1.0) [16,17,18]. Nonetheless, continued incubation of sediment slurries coupled with methane consumption resulted in highly elevated extractable levels of three hopanoids (aminotriol, aminotetrol and aminopenol) as well as two C-3 methylated hopanoids (3-methylaminotriol and 3-methylaminotetrol) that were enriched ≥1000-fold over the un-incubated sediment (Table 2; Figure 3). This assemblage of hopanoids occurs specifically in methanotrophs found within the *Gammaproteobacteria* [53]. The *pmoA* results further underscore this finding (Figure 4), while the results with 16S rRNA gene sequences indicate contributions from both the *Gamma*- as well as the *Alphaproteobacteria* (Figure 5 and Figure 6). We do not, of course, rule out the possible additional presence of other types of methanotrophs that may have eluded our molecular detection protocols.

Sustained cultivation of the methanotrophs from Herman Pit in liquid medium proved problematic once serial transfers made over time diluted out the initial sediment component. We had only modest success with adding small amounts of sterilized clay minerals to tubes in that the microorganisms would eventually fail to grow after only one or two sequential transfers. Only by inclusion of some of the original sediment material could we manage to achieve notable methane oxidation and growth, yet even in these cases the organisms proved capricious, achieving growth in some transfer tubes but not others that were inoculated simultaneously. It is possible that some essential but unidentified growth factor, perhaps rare earths [63], cobalamin [64], or some other factor provided by heterotrophs [65] was missing from the media we employed. Future research effort is required to establish better means to sustain methanotrophic enrichments and isolate pure cultures from the Herman Pit system.

Considering the fact that the Herman Pit is a former mercury cinnabar mine, it would be of interest to determine in the future if the resident methanotrophs play some role in the cycling of mercury in this contaminated environment. For example, the formation and destruction of monomethyl mercury (CH_3_Hg^+^) is often a focus of such investigations. The well-studied process of demethylation is usually carried out by an organo-mercurial lyase (*merB*), which cleaves off the methyl group to form methane, while a mercuric reductase (*merA*) reduces the Hg^2+^ to volatile Hg^0^. Demethylation could also be achieved by a process termed “oxidative demethylation” whereby the microbes involved attack the methyl group by recognizing CH_3_Hg^+^ as a C_1_ substrate (e.g., methanol, methylamine) rather than as a toxic material needed to be rendered harmless to the cells. Oxidative demethylation has been noted in previous studies made with a variety of sediments, mostly anoxic, and with cultures of sulfate reducers and methanogens [66,67,68,69], but the possible involvement of aerobic methanotrophs has not been investigated. Boden and Murrell [70] reported that *Methylococcus capsulatus* (Bath), a member of the *Gammaproteobacteria*, was able to channel electrons derived from methane oxidation into reduction of externally added Hg^2+^ and that a *mer*-mediated pathway appeared involved. They did not, however, investigate the culture’s ability to metabolize CH_3_Hg^+^, as they only followed the fate of ^14^CO_2_ and ^14^C-labelled cells as produced from ^14^CH_4_ oxidation in the presence or absence of added 10 mM HgCl_2_. However, considering the common occurrence of aerobic methanotrophs in nature, coupled with their ability to detoxify Hg^2+^ by chelation with methanobactin [71] or to carry out its reduction to Hg^0^ [70], a closer investigation of their role in the biogeochemical cycle or mercury is warranted. It would be of interest to determine if the Herman Pit organisms can effect oxidative demethylation of CH_3_Hg^+^, and if so, is oxidative demethylation a commonality amongst aerobic methanotrophs and methylotrophs. The latter possibility would suggest a broader involvement of C_1_-metabolizing aerobes in the cycle of methylated toxic metals, such as mercury, then is currently appreciated.

## 5. Conclusions

Ebullition of methane rich gases in the acidic environment of the Herman Pit, a former cinnabar mine, provides a sedimentary environment from which methane-oxidizing bacteria can be cultivated. Prolonged aerobic incubation of sediment slurries with methane resulted in notable increases in the recoverable sterols and hopanoid lipids characteristic of methanotrophs of the *Gammaproteobacteria*. These results were underscored by successful amplification of *pmmoA* sequences that aligned within that clade, although results with 16S rRNA sequences suggested a contributing component of methanotrophs from the *Alphaproteobacteria* as well. The sediments themselves showed optimal methane-oxidation activity at a mildly acidic pH (4.5). Cultivation of enrichments in mineral salts medium proved challenging in that a stable cultivar could not be established that could successfully be carried beyond two transfers unless given a small amount of the methane-enriched sediment. Hence, isolation of a pure culture of a methanotroph on a defined mineral salts medium from this locale was not achieved.
